# Joint conditional generative adversarial networks for eyelash artifact removal in ultra-wide-field fundus images

**DOI:** 10.3389/fcell.2023.1181305

**Published:** 2023-05-05

**Authors:** Jiong Zhang, Dengfeng Sha, Yuhui Ma, Dan Zhang, Tao Tan, Xiayu Xu, Quanyong Yi, Yitian Zhao

**Affiliations:** ^1^ Cixi Institute of Biomedical Engineering, Ningbo Institute of Materials Technology and Engineering, Chinese Academy of Sciences, Ningbo, China; ^2^ The Affiliated Ningbo Eye Hospital of Wenzhou Medical University, Ningbo, China; ^3^ Faculty of Electrical Engineering and Computer Science, Ningbo University, Ningbo, China; ^4^ School of Cyber Science and Engineering, Ningbo University of Technology, Ningbo, China; ^5^ Faulty of Applied Sciences, Macao Polytechnic University, Macao, Macao SAR, China; ^6^ The Key Laboratory of Biomedical Information Engineering of Ministry of Education, School of Life Science and Technology, Xi’an Jiaotong University, Xi’an, China; ^7^ Zhejiang Research Institute of Xi’an Jiaotong University, Hangzhou, China

**Keywords:** retina, ultra-wide-field fundus images, artifact removal, conditional GAN, vessel segmentation

## Abstract

**Background:** Ultra-Wide-Field (UWF) fundus imaging is an essential diagnostic tool for identifying ophthalmologic diseases, as it captures detailed retinal structures within a wider field of view (FOV). However, the presence of eyelashes along the edge of the eyelids can cast shadows and obscure the view of fundus imaging, which hinders reliable interpretation and subsequent screening of fundus diseases. Despite its limitations, there are currently no effective methods or datasets available for removing eyelash artifacts from UWF fundus images. This research aims to develop an effective approach for eyelash artifact removal and thus improve the visual quality of UWF fundus images for accurate analysis and diagnosis.

**Methods:** To address this issue, we first constructed two UWF fundus datasets: the paired synthetic eyelashes (PSE) dataset and the unpaired real eyelashes (uPRE) dataset. Then we proposed a deep learning architecture called Joint Conditional Generative Adversarial Networks (JcGAN) to remove eyelash artifacts from UWF fundus images. JcGAN employs a shared generator with two discriminators for joint learning of both real and synthetic eyelash artifacts. Furthermore, we designed a background refinement module that refines background information and is trained with the generator in an end-to-end manner.

**Results:** Experimental results on both PSE and uPRE datasets demonstrate the superiority of the proposed JcGAN over several state-of-the-art deep learning approaches. Compared with the best existing method, JcGAN improves PSNR and SSIM by 4.82% and 0.23%, respectively. In addition, we also verified that eyelash artifact removal via JcGAN could significantly improve vessel segmentation performance in UWF fundus images. Assessment via vessel segmentation illustrates that the sensitivity, Dice coefficient and area under curve (AUC) of ResU-Net have respectively increased by 3.64%, 1.54%, and 1.43% after eyelash artifact removal using JcGAN.

**Conclusion:** The proposed JcGAN effectively removes eyelash artifacts in UWF images, resulting in improved visibility of retinal vessels. Our method can facilitate better processing and analysis of retinal vessels and has the potential to improve diagnostic outcomes.

## 1 Introduction

**FIGURE 1:**
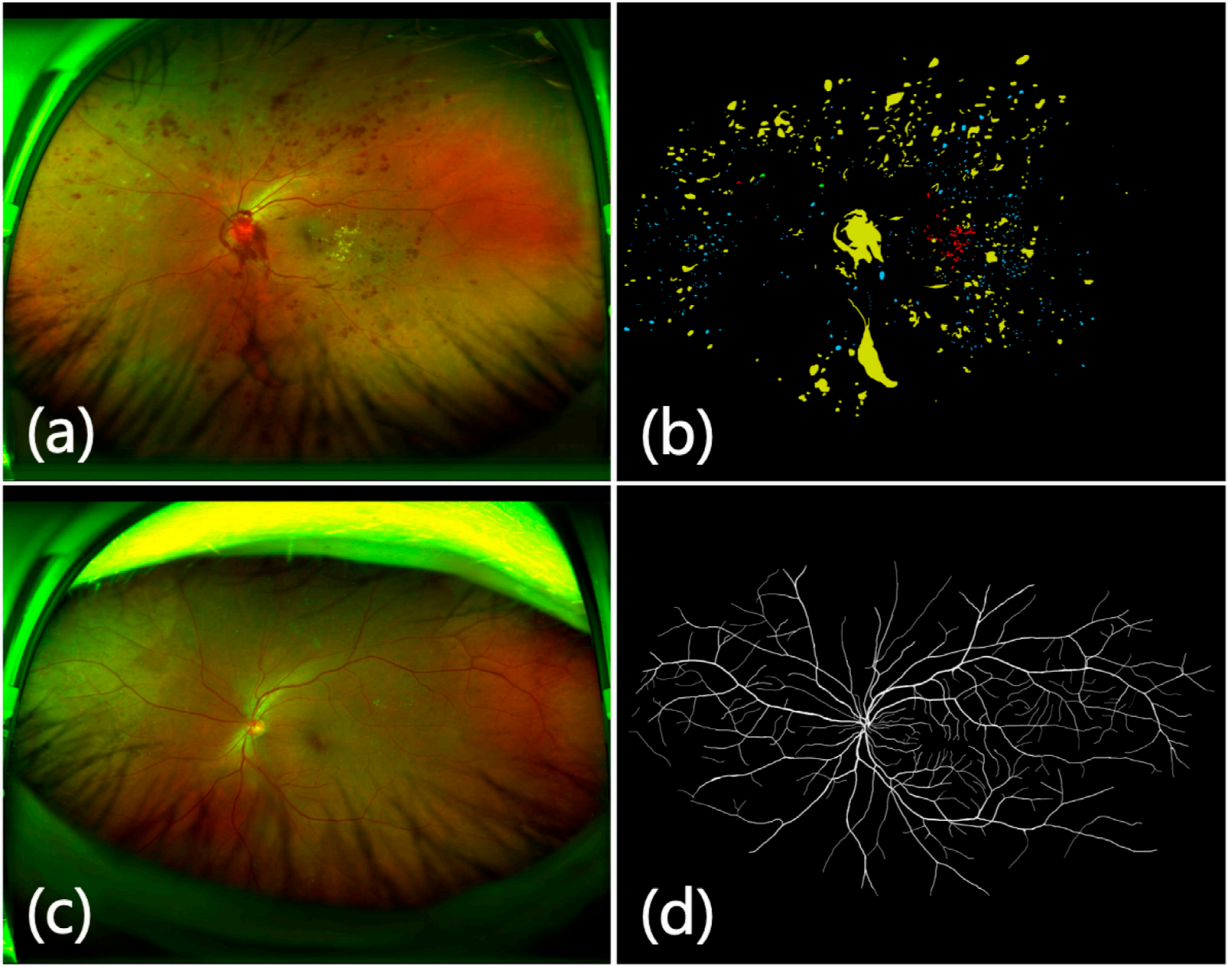
Detailed illustration of eyelash artifacts obscuring lesions and blood vessels. **(A)** The eyelash artifact obscures the lesion information. **(B)** Ground truth of the lesion in **(A)**. **(C)** The eyelash artifact obscures the vessel information. **(D)** Ground truth of the vessel in **(C)**

Ultra-Wide-Field (UWF) fundus images are a new type of retinal colour fundus image with ultra wide angle characteristics, which can cover 200° Patel et al. (2020) of the retinal fundus in a single image. It has significant advantages over conventional colour fundus images in screening and detecting retina-related diseases such as diabetic retinopathy. However, the imaging characteristics of UWF fundus images often lead to problems with eyelash artefacts in UWF fundus images. As shown in Figure 1, eyelash artefacts obscure the site of the lesion and some of the blood vessels, making it difficult to clearly distinguish key information. In the diagnosis of clinical disease, eyelash artefacts are a serious problem in terms of image quality and pose a significant diagnostic challenge to physicians Kornberg et al. (2016); Ajlan et al. (2020).

To reduce the effect of eyelash artifacts, some physical methods are often applied to the UWF imaging acquisition. These methods include manually pulling up the eyelid, retracting eyelashes via cotton bud Cheng et al. (2008), holding down eyelashes via disposable eyelid speculum (EzSpec) Inoue et al. (2013) and expanding eyelids with the eyelid clamper Ozawa et al. (2020), etc. Although these methods can reduce the appearance of eyelash artifacts to a certain extent, they are not able to completely solve the problem of eyelash artifacts, and these methods bring new challenges during surgical inspections Inoue et al. (2013). Therefore, eyelash artifact has always plagued doctors as a problem with the interpretation of UWF images. In recent years, researchers have found that eyelash artifact is a serious interference problem in the study of UWF images such as lesion detection and blood vessel segmentation Yoo et al. (2020); Li et al. (2020, 2019), as shown in Figure 1. Given the adverse effects of eyelash artifacts on both clinical diagnosis and computer vision tasks, it is necessary to develop an automatic and effective method for removing eyelash artifacts from UWF images.

To the best of our knowledge, there is no automatic algorithm that has been proposed for eyelash artifact removal of UWF images. The main reason is that it is difficult to obtain corresponding image pairs eyelashes/eyelashes-free, and super-sized images are very important for model design and training strategy is no small challenge. At present, the task of removing shadow occlusion Fan et al. (2019) in natural image processing is similar to the task of removing eyelash artifacts in UWF images, both of which are dedicated to removing occlusion artifacts and recovering occluded information Chen et al. (2021). However, compared with natural images, it is more difficult to remove eyelash artifacts in UWF images Matsui et al. (2019). For example, the features of eyelash artifacts are complex and diverse, with large differences, and the structures of blood vessels and lesions are relatively small. Hence, the difficulties of fully automatic UWF image eyelash removal methods: On the one hand, relying on an image acquisition process like natural images, it is impossible to obtain paired UWF images (i.e., images with eyelashes and corresponding eyelash-free labels) for supervised learning. On the other hand, eyelash artifacts in UWF images are usually highly complex and diverse, which makes it difficult to preserve some fine structures such as blood vessels/lesions in the eyelash artifact area for further analysis. Most of the UWF images currently available contain eyelashes, only a small part contains no eyelashes at all and a few contain few eyelashes, and there are no matching image pairs of eyelashes/eyelashes-free at all. Secondly, when designing the model, it is necessary to take into account the removal of eyelashes and the recovery of the information occluded by the eyelashes Mackenzie et al. (2007), and what method to use for training large-size images is also a problem that needs to be considered.

**FIGURE 2:**
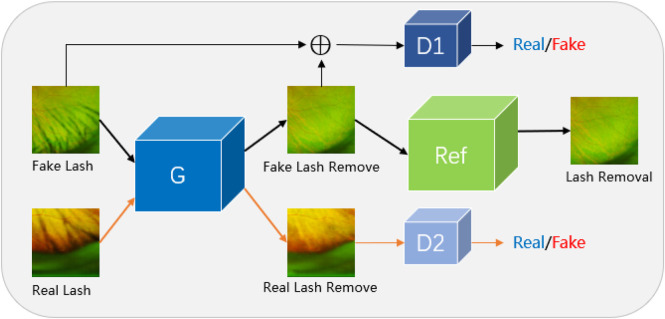
We propose Joint Conditional Generative Adversarial Networks (JcGAN) for eyelash artifact removal from UWF images. The network includes a generator G, two discriminators D1 and D2 and a background refinement module called Ref. The generator G and the discriminator D1 form a conditional generative adversarial network that takes the synthetic eyelashes (SEL) dataset as input. The generator G and the discriminator D2 form a generative adversarial network that takes the real eyelashes (REL) dataset as input.

In response to the problems raised above, this paper proposes a Joint Conditional Generative Adversarial Network (JcGAN) to remove eyelash artifacts from UWF images and constructs to two UWF image datasets: synthetic eyelashes (SEL) and real eyelashes (REL). The joint conditional generative adversarial network (See Figure 2) adopts the combination of conditional adversarial network and adversarial network and uses two sets of data sets as input to train the same generator, which not only trains the generator to remove synthetic eyelashes but also trains the generator to remove real eyelashes ability. Connect a background refinement module after the generator to ensure background integrity.

The proposed method extends considerably our previous work Sha et al. (2022), which was trained only on the paired samples with synthetic eyelash artifacts generated from the proposed Eyelash Growing Model. In this work, we first extended our synthetic dataset in a manner contrary to Eyelash Growing Model, where paired samples were obtained by manually erasing eyelash artifacts from real UWF images. Secondly, we have collected an unpaired dataset, which consists of real UWF images with and without eyelash artifacts. In order to fully utilize the unpaired samples and thus further enhance the generalization performance on real UWF images with eyelash artifacts, we have also improved the architecture by introducing one additional discriminator into the generative adversarial network, which shares the generator with the original one. Different from the original discriminator, the additional discriminator aims at distinguishing between real samples without eyelash artifacts and the ones generated from real samples with eyelash artifacts. To this end, the additional discriminator could constrain the generator to improve the performance of eyelash artifact removal on the real UWF images. Overall, the contributions of our work can be summarized as follows:

• For the first time in the UWF fundus imaging field, we construct two datasets for eyelash artifact removal, which respectively consist of paired images with/without synthetic eyelash artifacts and unpaired images with/without real eyelash artifacts.

• We develop a deep learning architecture called Joint conditional Generative Adversarial Networks (JcGAN), which adopts one shared generator with two discriminators for jointly removing real and synthetic eyelash artifacts and utilizes a background refinement module to refine background information.

• We apply the proposed JcGAN on the datasets for eyelash artifact removal. Both quantitative and qualitative results demonstrate the superiority of the proposed JcGAN in eliminating eyelash artifacts and its performance gains to the vessel segmentation task.

## 2 Related works

### 2.1 GAN and CGAN

Generative Adversarial Network (GAN) was first proposed by Ian Goodfellow Goodfellow et al. (2014). It is a framework for estimating generative models through an adversarial process, including a generative model G that captures data distribution and a discriminant model D that estimates the probability that samples come from training data rather than G-generated data. The training goal of generative model G is to generate images similar to the target domain to greatly increase the error probability of discriminant model D, while the training goal of discriminant model D is to greatly reduce the probability of discriminatory errors. A minimax game process is the so-called generative confrontation. The generative adversarial model is only a mapping from the source domain to the target domain, and cannot specify a fixed target, which is caused by the lack of target guidance. Conditional generative adversarial network (CGAN) Mirza and Osindero (2014) is to add prior conditions to both the generator and the discriminator based on the generative adversarial network, so that a conditional model is formed into the guidance of additional conditions. This extra condition is diverse, it can be class labels or other patterns of data, guided by the extra condition, we can generate a fixed single target for the generator.

### 2.2 Eyelash artifact removal from UWF images

Since the availability of UWF images, the range of fundus examinations has been greatly improved and the efficiency of fundus screening has been increased, providing an efficient method of screening for a wide range of eye diseases. However, the existence of eyelash artifacts has increased the difficulty of automatic UWF image examination. At present, methods of removing eyelash artifacts from UWF images are limited to physical avoidance methods of the shooting process. Inoue et al. (2013) have invented a disposable eyelid mirror (EzSpec), a flexible translucent speculum that keeps the eye open to misalignment and covers a wider eyelash area, but the use process requires topical anesthesia, which is expensive and not universal. Ozawa et al. (2020) invented an eyelid clamp to circumvent the problem of eyelash artifacts during UWF images to capture. It is a face-worn tool that keeps the eyes open by applying pressure in the eyelid area, but the avoidance effect of eyelash artifacts is not obvious. In addition, there are some small ways to avoid eyelashes in the process of taking UWF images, such as using tape to stick eyelashes, using cotton swabs to converge eyelashes, or pulling up eyelashes directly by hand, etc. However, these methods The effect of avoiding eyelashes is not obvious, and it is not easy to operate and control. Therefore, the problem of eyelash artifacts in UWF images has always been a disturbing factor of UWF images.

### 2.3 Shadow removal

The problem of eyelash artifact occlusion in UWF images is similar to the problem of shadow removal of natural images. However, the automatic algorithm for eyelash artifact removal of UWF images has not been studied before, while the automatic removal algorithms for the task of natural image shadow removal have been extensively explored. In general, image shadow removal algorithms can be divided into traditional methods and deep learning based methods. The traditional methods were developed based on image gradient Finlayson et al. (2005); Gryka et al. (2015), lighting information Yang et al. (2012); Zhang et al. (2015), and region attributes Guo et al. (2012). Deep learning based methods mainly include supervised learning models Zhang et al. (2019); Liu et al. (2020) and unsupervised learning models Hu et al. (2019b).

Previous methods remove shadows by modeling the image as a combination of shadow and shadow-free components Arbel and Hel-Or (2010); Finlayson et al. (2009, 2002), or by shifting colors from shadow-free to shadow regions Shor and Lischinski (2008); Wu and Tang (2005); Xiao et al. (2013). Due to the limitations of the underlying models in those methods, they are usually unable to handle shadows in complex real-world scenes Khan et al. (2015). Following that, researchers explored statistical modeling methods to discover and remove shadows using features such as intensity Gong and Cosker (2014), color Guo et al. (2012), texture Khan et al. (2014), and gradient Finlayson et al. (2005); Gryka et al. (2015). However, these handcrafted features are hard to represent the complex features of shadows. Therefore, Khan et al. (2015) propose a method of using a convolutional neural network (CNN) to detect shadows and then using a Bayesian model to remove shadows. Qu et al. (2017) develop three sub-networks to extract features of multiple views separately, and embedded all sub-networks into a complete framework for shadow removal. Wang et al. (2018) used one conditional generative adversarial network (CGAN) to detect shadows and another CGAN to remove shadows. Hu et al. (2019a) explore orientation-aware spatial context methods to detect and remove shadows. However, these methods are trained in paired images, which are limited by paired datasets. To get rid of the dependence on paired data, Hu et al. (2019b) propose a Mask Shadow GAN framework based on Cycle GAN Zhu et al. (2017), which utilizes unpaired data to learn the mapping from unshadowed domains to shadowed domains and *vice versa* Of course. Later Liu et al. (2021) develop the LG Shadow Net framework to improve the Mask Shadow GAN Hu et al. (2019b) by introducing a brightness-guided strategy that uses the learned brightness features to guide the learning of shadow removal.

## 3 Datasets

To the best of our knowledge, there is no research using deep learning methods for eyelash artifact removal of UWF images. Also, there is no publicly available dataset on eyelash artifacts in UWF images. This paper constructs two new datasets of eyelash artifacts in UWF images. The first is the paired synthetic eyelashes (PSE) dataset and the second are the unpaired real eyelashes (uPRE) dataset. Table 1 presents the details of the two datasets. All data used in this paper were collected from the affiliated Ningbo Eye Hospital of Wenzhou Medical University and Ningbo People’s Hospital at Ningbo, China. The acquisition device was an Optos fundus camera (Optos PLC, Dunfermline, Scotland). Prior to examination, written informed consents were obtained from subjects in accordance to the tenets of Declaration of Helsinki. The PSE dataset consists of 7025 pairs of eyelash and eyelash-free images with a size of 1024 × 1024, where the eyelashes are from the eyelash growing model Sha et al. (2022). We used 5975 pairs of images as the training set and 1050 pairs of images as the test set. The uPRE dataset includes 3687 each of eyelash images and eyelash-free images with a size of 1024 × 1024, where the eyelashes are from the patients themselves. We used 3037 pairs of images as the training set and 650 pairs of images as the test set.

**TABLE 1:** Details of the two datasets PSE and uPRE.

Datasets	Amount	Content of images	Type
PSE	7025	eyelash/eyelash-free	pair
uPRE	3687	eyelash/eyelash-free	unpair

### 3.1 Paired synthetic eyelashes dataset

In practice, it is difficult to obtain paired eyelash/eyelash-free UWF images by controlling the eyelash variables during image acquisition, as is in the case of ISTD Wang et al. (2018). Previously, we proposed an eyelash growing model in the DelashNet Sha et al. (2022) method to solve the above problem. Since the lash removal performance can be easily affected by the reliability of the eyelash growing model, we additionally set up more realistic data pairs into the training set to better guide the model. To this end, we respectively adopted forward and reverse synthesis methods to generate the pairwise dataset for eyelash artifact removal. For the forward synthesis method, the eyelash growing model was developed to simulate eyelash features and generate synthetic eyelashes, followed by a fusion procedure to combine eyelash-free UWF images. For the reverse synthesis method, *Photoshop* is used to manually erase eyelash artifacts from UWF images and thus generate eyelash-free images. The forward synthesis method fails to simulate the complicated characteristics of eyelash artifacts, thus hinders the model’s capability of identifying and eliminating real eyelash artifacts. Conversely, the reverse synthesis method preserves the authenticity of the eyelash artifacts, but this process may distort the background. The paired data generated in the above two ways construct the Paired Synthetic Eyelashes (PSE) Dataset in this work.

### 3.2 Unpaired real eyelashes dataset

A UWF image contains both eyelash information and eyelash-free information. Therefore, UWF image patches with eyelashes and without eyelashes can be separately obtained by cropping the entire image. We cropped the large size (3900 × 3072) UWF images into several small-size patches for training. The cropped patch size is also an important issue that needs to be considered. If the size is too small, less global information can be preserved. While if the size is too large, it will be impossible to achieve high computational efficiency. Therefore, we finally use patch size of 1024 × 1024 for data training. Although the data with eyelashes and without eyelashes are not completely matched, the construction basis of this real data set is still of great significance to our follow-up model design.

## 4 Proposed method

**FIGURE 3:**
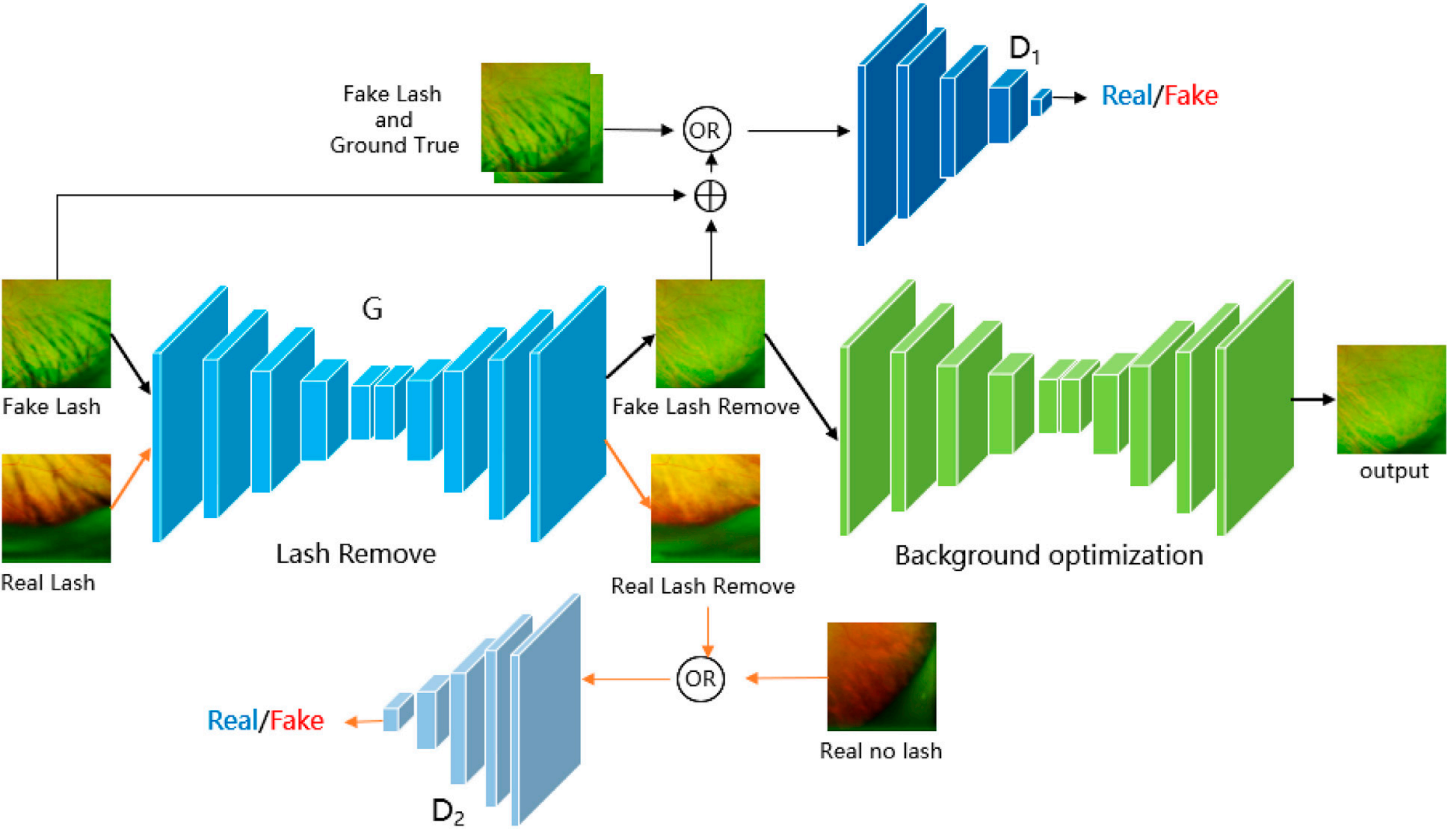
Illustration of the architecture of our proposed JcGAN-Net.

In this section, we introduce the proposed architecture called JcGAN, for eyelash artifact removal in UWF fundus images. The overall framework of JcGAN is illustrated in Figure 3. It adopts one shared generator with two discriminators and learns to translate those images with eyelash artifacts into artifact-free ones via adversarial training jointly with paired and unpaired samples. JcGAN also introduce an additional background refinement module into an end-to-end process, in order to further restore background information obscured by eyelash artifacts.

### 4.1 Architecture

Our JcGAN consists of two generative adversarial networks with one shared generator *G* and an addtional background refinement module (BRM), as shown in Figure 3. The generator *G* tries to generate the corresponding artifact-free image from the input image with synthetic or real eyelash artifacts, and the discriminator *D*_1_ (*D*_2_) attempts to distinguish between real artifact-free images and the ones generated from synthetic (real) samples. In order to further restore background details covered by eyelash artifacts, the background refinement module (BRM) is applied to refine the generated results from the generator *G* via end-to-end training.

Both the generator *G* and background refinement module (BRM) adopts the same U-shape structure, which contains eight encoder-decoder layers with symmetric skip connections Ronneberger et al. (2015). All encoder layers employ 4 × 4 convolution with stride 2 followed by Batch Normalization (BN) and Leaky ReLU, except the last encoder layer with ReLU instead and no BN. For the first seven decoder layers, we utilize 4 × 4 transposed convolution with stride 2 followed by BN and ReLU. The last decoder layer also removes BN and outputs the final result through Tanh function.

For both discriminators *D*_1_ and *D*_2_, we construct a network with five 4 × 4 convolutional layers, where stride is set to 2 in the first three layers and 1 in the last two layers. BN is used in the 2*nd*-4*th* layers. All layers introduce Leaky ReLU except the last layer. Finally, the discriminator network outputs a confidence map via Sigmoid function, where each pixel represents the probability that the corresponding local region of the input image is identified as coming from a real artifact-free sample.

### 4.2 Loss function

In order to effectively constrain the proposed JcGAN, we employ the joint adversarial training strategy to optimize the architecture end-to-end based on both paired and unpaired samples. Finally, we construct the loss function including conditional adversarial loss, unconditional adversarial loss and refinement loss.

• Conditional adversarial loss For a synthetic pair of corruption/artifact-free samples (*x*_*p*_/*y*_*p*_), the generator *G* takes *x*_*p*_ and random noise vector *z* as input and attempts to produce the fake result (denoted as *G* (*z*, *x*_*p*_)) which is close to *y*_*p*_ as possible, while the discriminator *D*_1_ attempts to classify between the real pair (*x*_*p*_, *y*_*p*_) and the fake pair (*x*_*p*_, *G* (*z*, *x*_*p*_)). Through the competition between *G* and *D*_1_, JcGAN can learn the mapping from corruption images to the corresponding artifact-free ones. Thus the conditional adversarial loss $L_{ca}$ can be expressed as:

$$\begin{array}{c} L_{ca}\left(G,D_{1}\right)=E_{x_{p},y_{p}∼p_{PSE}\left(x_{p},y_{p}\right)}\left[\log D_{1}\left(x_{p},y_{p}\right)\right]\\ +E_{x_{p}∼p_{PSE}\left(x_{p}\right),z∼p_{z}\left(z\right)}\left[\log\left(1-D_{1}\left(x_{p},G\left(z,x_{p}\right)\right)\right.\right] \end{array}$$ (1)

In addition, we also introduce L1 distance to further minimize the discrepancy between the generated image *G* (*z*, *x*_*p*_) and the real artifact-free image *y*_*p*_:

$$L_{1}\left(G\right)=E_{x_{p},y_{p}∼p_{PSE}\left(x_{p},y_{p}\right),z∼p_{z}\left(z\right)}\|y_{p}-G\left(z,x_{p}\right){\|}_{1}$$ (2)

• Unconditional adversarial loss For unpaired corruption/artifact-free samples (*x*_*u*_/*y*_*u*_), the generator *G* also takes *x*_*u*_ as input and attempts to produce the fake result (denoted as *G* (*z*, *x*_*u*_)), while the discriminator *D*_2_ attempts to identify whether one given image is real or fake artifact-free image. The competition between *G* and *D*_2_ could promote the perceptual quality of generated images from *G*. Therefore, the unconditional adversarial loss $L_{uca}$ can be denoted as:

$$\begin{array}{c} L_{uca}\left(G,D_{2}\right)=E_{y_{u}∼p_{uPRE}\left(y_{u}\right)}\left[\log D_{2}\left(y_{u}\right)\right]\\ +E_{x_{u}∼p_{uPRE}\left(x_{u}\right),z∼p_{z}\left(z\right)}\left[\log\left(1-D_{2}\left(G\left(z,x_{u}\right)\right)\right.\right] \end{array}$$ (3)

• Refinement loss In order to constrain background refinement module (denoted as *R*) to produce refined artifact-free results more precisely, we adopt L1 distance as refinement loss:

$$L_{ref}\left(G,R\right)=E_{x_{p},y_{p}∼p_{PSE}\left(x_{p},y_{p}\right),z∼p_{z}\left(z\right)}\|y_{p}-R\left(G\left(z,x_{p}\right)\right){\|}_{1}$$ (4)

Finally, the total loss function of the proposed JcGAN is defined as:

$$\begin{array}{c} L_{total}\left(G,D_{1},D_{2},R\right)=L_{ca}\left(G,D_{1}\right)+L_{1}\left(G\right)\\ +L_{uca}\left(G,D_{2}\right)+L_{ref}\left(G,R\right) \end{array}$$ (5)

where *λ*_1_ and *λ*_2_ represent the weighted parameters of L1 distance and refinement loss.

## 5 Experimental setup

In this section, we describe the experimental setups, including the evaluation metrics, data ablation, module ablation and comparative experiments.

### 5.1 Implementation settings

The proposed JcGAN was implemented with PyTorch library, and the experiments were conducted on two NVIDIA GPUs (Tesla V100 with 32 GB). All training images were resized to 1024 × 1024, and a random horizontal flipping was applied for data augmentation. Adam optimization was applied to train the model, with epochs of 200, the initial learning rate of 0.0002 and batch size of 15. The weighted parameters in the final objective function were experimentally set as: *λ*_1_ = 100 and *λ*_2_ = 10.

### 5.2 Evaluation criteria

We verify the synthesic data and real data separately. For synthesic paired data, the traditional image enhancement Maini and Aggarwal (2010) evaluation criteria are used to calculate PSNR and SSIM Hore and Ziou (2010):

• Peak Signal to Noise Ratio (*PSNR*);

$$\begin{array}{c} PSNR=10\times \log\left(\frac{{\left(2^{n}-1\right)}^{2}}{MSE}\right) \end{array}$$ (6)

where *MSE* Sara et al. (2019) is the mean squared error between the original image and the processed image.

• Structural Similarity (SSIM);

$$\begin{array}{c} SSIM\left(x,y\right)=\frac{\left(2\mu_{x}\mu_{y}+c_{1}\right)\left(2\delta_{xy}+c_{2}\right)}{\left(\mu_{x}^{2}+\mu_{y}^{2}+c_{1}\right)\left(\delta_{x}^{2}+\delta_{y}^{2}+c_{2}\right)} \end{array}$$ (7)

where *μ*_*x*_ is the mean of *x*, *μ*_*y*_ is the mean of *y*, *δ*_*x*_ is the variance of *x*, *δ*_*y*_ is the variance of *y*, and *δ*_*xy*_ is the covariance of *x* and *y*. $c_{1}={\left(\kappa_{1}L\right)}^{2}$ , $c_{2}={\left(\kappa_{2}L\right)}^{2}$ is a constant used to maintain stability. *L* is the dynamic range of pixel values. *κ*_1_ = 0.01, *κ*_2_ = 0.02. Structural similarity ranges from −1 to 1. When the two images are identical, the value of SSIM is equal to 1.For the real unpaired data, we use the equivalent numbers of looks in the local area to evaluate the smoothness of the processed image. Additionally, we use the performance on the validation vessel segmentation task as an indirect evaluation metric.

• Equivalent numbers of looks (*ENL*) Vespe and Greidanus (2012);

$$\begin{array}{c} ENL=\frac{\mu^{2}}{\delta^{2}} \end{array}$$ (8)

where *μ* is the mean of the local area of the image, *δ* is the variance of the local area of the image. ENL is commonly used to measure the speckle suppression of different SAR/OCT image filters. When the ENL value is bigger, it indicates the image is smoothed well.

• Resunet was used to train a vessel segmentation network, which was indirectly validated by the effect on vessel segmentation performance before and after eyelash artifact removal.

### 5.3 Data ablation

As mentioned above, two datasets including PSE and uPRE are used for evaluation. The PSE dataset consists of two parts, PSE part 1 (PSE1) from the eyelash growing model and PSE part 2 (PSE2) from manual erasure. PSE1 is characterized by the fact that the synthetic eyelashes can only approximate the key information of the real eyelashes to some extent, but cannot completely model the real eyelashes. PSE2 is used to compensate PSE1 by including paired eyelash information from realstic UWF images. To verify the effectiveness of the two data generation approaches, we conduct the data ablation experiments as follows. We designed three experiments to verify the performance of the three dataset combinations respectively. (i) The combination of PSE1 dataset and uPRE dataset. (ii) The combination of PSE2 dataset and uPRE dataset. (iii) The combination of PSE dataset and uPRE dataset.

**FIGURE 4:**
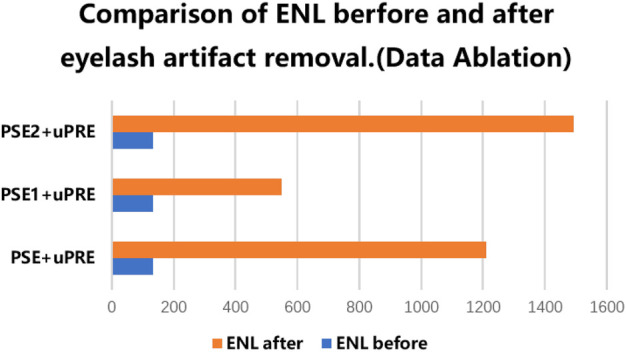
Comparison of ENL before and after the real eyelash artifact removal, where the blue bar represents the ENL value of the eyelash artifact area before eyelash artifact removal, and the orange bar represents the ENL value of the same area after eyelash artifact removal.

We used the data of the above three combinations to train three models. For each model, we also tested the three sets of data: PSE1, PSE2 and PSE. Therefore, in the experiment of data ablation, we have completed a total of 3 × 3 data testing. Table 2 shows the values of the PSNR and SSIM of the 3 × 3 groups. The results show that the training method using the third data combination achieves the best results. The results show that the model trained on the PSE + uPRE data achieves the best results. First of all, the PSE1 does not simulate all the information of real eyelashes. Adding the PSE2 reduce the effects of lacking of synthetic eyelashes. Second, the ground truth from PSE2 data has limitations with inaccurate backgrounds. Adding accurate ground truth from the PSE1 can compensate this issue in the PSE2. At the same time, we use the uPRE dataset to verify the effects of the three models. As shown in Figure 4, the ENL results of the real data have been improved to a certain extent.

**TABLE 2:** The values of the PSNR and SSIM tests of our 3×3 groups.

Methods	PSNR			SSIM		
	PSE1	PSE2	PSE	PSE1	PSE2	PSE
PSE1+uPRE	39.270	37.488	40.125	0.9640	0.9304	0.9585
PSE2+uPRE	35.935	**38.972**	38.073	0.9475	0.9253	0.9462
PSE + uPRE	40.182	38.943	43.692	0.9652	0.9353	0.9729

As shown in Figure 4, all three different data resulted in improved ENL after eyelash removal, among which the combination of PSE2+uPRE achieved the largest improvement, and the combination of PSE + uPRE achieved the second rank. For the test results of synthetic eyelashes data, the combination of PSE + uPRE achieved the best results, which met our expectations. While for the test results of the real eyelash data, the combination of PSE + uPRE has not achieved the best results in the test of real eyelash data. We know that ENL only calculates the local area of eyelash artifact. Therefore, in order to fully verify the performance of these three sets of data, it is necessary to compare them in a larger area.

**FIGURE 5:**
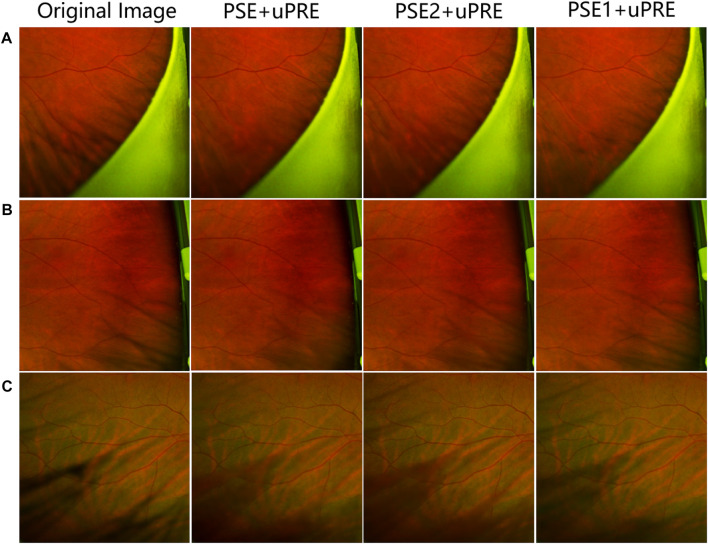
Visual representation of a data ablation experiment.**(A–C)** represent three different pictures, the first column shows the original picture, the second column shows the test results of PSE + uPRE training, and the third column shows the test results of PSE2 + uPRE training, the fourth column shows the test results of the PSE1 + uPRE training.

Figure 5 shows the test results on three sets of training data. From the figure, we can see that the results of the PSE + uPRE training data are significantly better than the results of the other two groups. It removes most of the artifacts and preserves the background much better. Thus, we take the PSE1+PSE2 data as the final PSE dataset.

### 5.4 Module ablation

The JcGAN proposed in this paper includes three sub-nets: a conditional generative adversarial Mirza and Osindero (2014) sub-net (cGAN-sub) composed of generator G and discriminator D1, a generative adversarial Goodfellow et al. (2014) sub-net (GAN-sub) composed of generator G and discriminator D2, and a background refinement sub-net (Ref-sub). To verify the contributions of each sub-net to the overall JcGAN network, we design module ablation experiments as follows.

According to the combination of different sub-net, we conduct a total of four module ablation experiments. 1) The conditional generative adversarial sub-net (cGAN-sub) is used as the baseline of the JcGAN network framework. Hence, we first design experiments to train the conditional generative adversarial sub-net to verify the effectiveness of the baseline module. 2) Based on the conditional generative adversarial sub-net (cGAN-sub), we separately add the background refinement sub-net (Ref-sub) to verify the utility of the background refinement sub-net on model performance. 3) Based on the conditional generative adversarial sub-net (cGAN-sub), we separately add the generative adversarial sub-net (GAN-sub) to verify the utility of the generative adversarial sub-net on model performance. 4) Finally, we add a generative adversarial sub-net (GAN-sub) and a background refinement sub-net (Ref-sub) on the baseline, i.e., our complete JcGAN network framework, to verify the effectiveness of all sub-networks.

After completing the above four experiments, we use the PSE dataset and the uPRE dataset to verify the results respectively. Table 3 shows the PSNR and SSIM values on the PSE dataset.

**TABLE 3:** The values of PSNR and SSIM tested on the PSE dataset for the module ablation experiments.

Methods	PSNR	SSIM
cGAN-sub	39.1357	0.9519
cGAN-sub + Ref-sub	42.1008	0.9640
cGAN-sub + GAN-sub	40.8884	0.9697
JcGAN	43.6922	0.9729

It is obvious from Table 3 that our method achieves competitive performance on the PSE dataset. The baseline of our model (cGAN-sub) has achieved significant breakthroughs in PSNR and SSIM values. The value of PSNR is as high as 39.1357, which is due to the high resolution Takahashi et al. (2019) of UWF images. Initially, we design Ref-sub as a background refinement sub-network in the overall framework of JcGAN, in order to ensure that the background occluded Audet and Cooperstock (2007) by eyelash artifacts can be fully recovered while eyelash artifacts are removed. Now, after adding Ref-sub on the basis of cGAN-sub, the values of PSNR and SSIM are further improved, which shows that Ref-sub plays an active role. After verifying the effectiveness of cGAN-sub and Ref-sub, we further verify the effectiveness of GAN-sub. Adding GAN-sub on the basis of cGAN-sub means that the joint idea of our JcGAN network is applied. The two datasets train the same generator alternatively so that this generator has the ability to remove synthetic eyelashes and real eyelashes. As shown in the results, our joint strategy achieve competitive performance on the PSE dataset. Finally, the test results of the JcGAN network also show that each sub-network in our whole framework plays an active role, and combining the three sub-networks can produce the best results.

**FIGURE 6:**
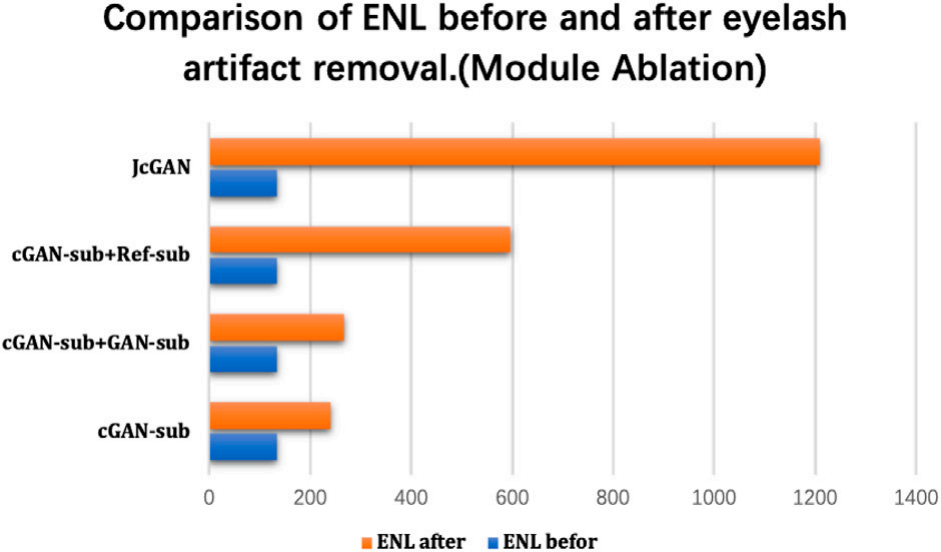
Comparison of ENL before and after the real eyelash artifact removal, where the blue bar represents the ENL value of the eyelash artifact area before eyelash artifact removal, and the orange bar represents the ENL value of the same area after eyelash artifact removal.

After being evaluated on the PSE Dataset, we also validate our method on the uPRE dataset. We used the local area of eyelash artifact removal to calculate the ENL value. Figure 6 shows the ENL values of the eyelash occluded area before and after eyelash artifact removal.

As shown in Figure 6, the combination of different sub-networks improves the value of ENL. In particular, the addition of the Ref-sub subnet has greatly improved the value of ENL. This shows that our Ref-sub sub-network effectively recovers the background of the eyelash artifact part. The JcGAN network framework improves the value of ENL the most, which also strongly proves that our joint strategy is also successful in the artifact removal of real eyelashes.

## 6 Discussion

### 6.1 Comparative analysis

To verify the effectiveness of our method, we selected several methods similar to ours for comparative experiments. Currently, no deep learning method has been proposed for artifact removal in ultra-widefield fundus images. Therefore, we selectively choose several classical GAN network related methods Pix2Pix Isola et al. (2017) and cycleGAN Zhu et al. (2017) and some natural image shadow removal methods ST-CGAN Wang et al. (2018) and Mask-ShadowGAN Hu et al. (2019b) as comparison methods in our analysis. We trained the above methods sequentially and tested each method using PSE dataset and uPRE dataset, as shown in Table 4.

**TABLE 4:** The values of PSNR and SSIM tested on the PSE Dataset for the comparative experiments.

Methods	PSNR	SSIM
Pix2Pix	34.1901	0.9281
Mask-ShadowGAN	37.2841	0.9462
CycleGAN	37.8026	0.9442
ST-CGAN	41.6814	0.9707
JcGAN	43.6922	0.9729

**FIGURE 7:**
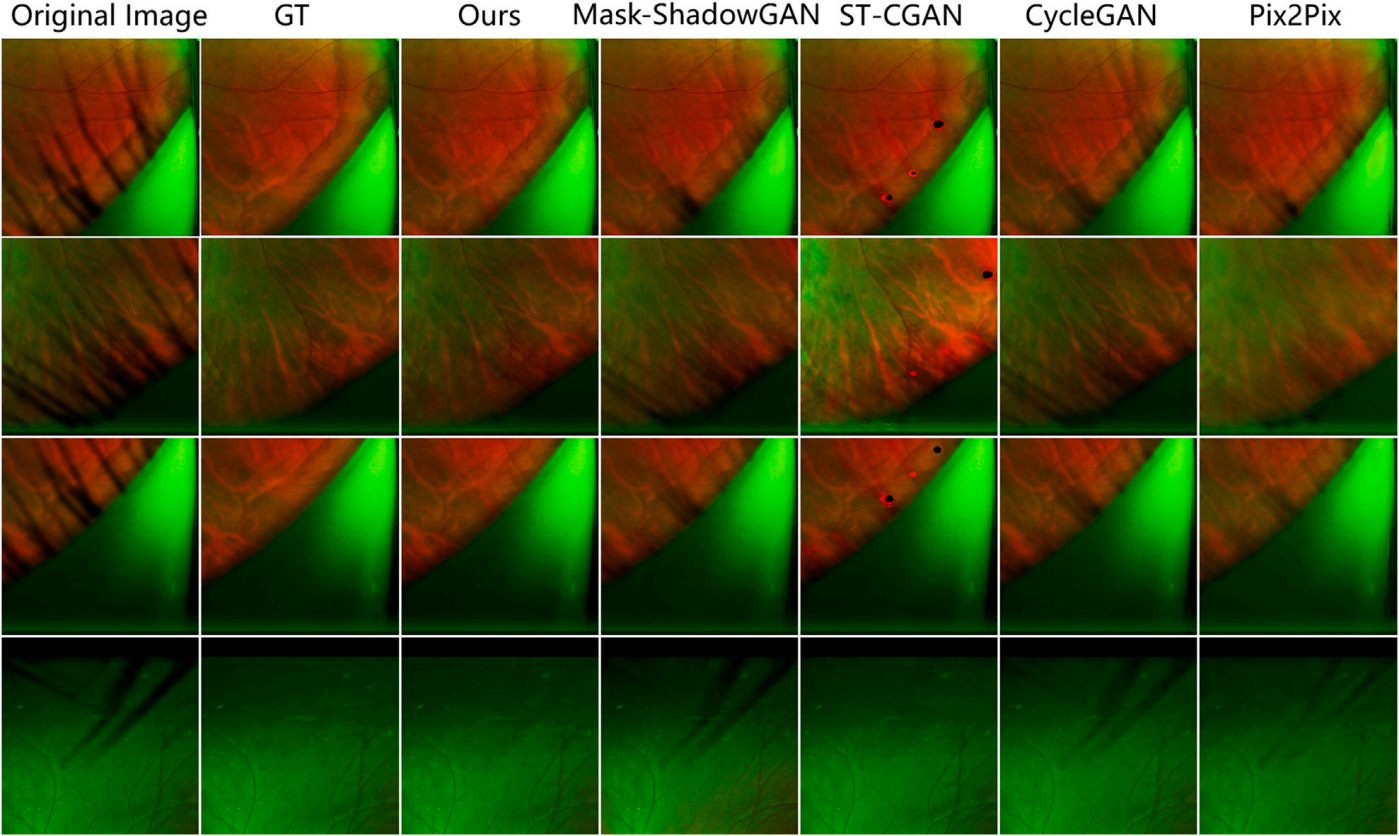
Comparison of the results of different methods on the PSE Dataset. Our method removes the most eyelash artifacts and restores the most realistic background information.

**FIGURE 8:**
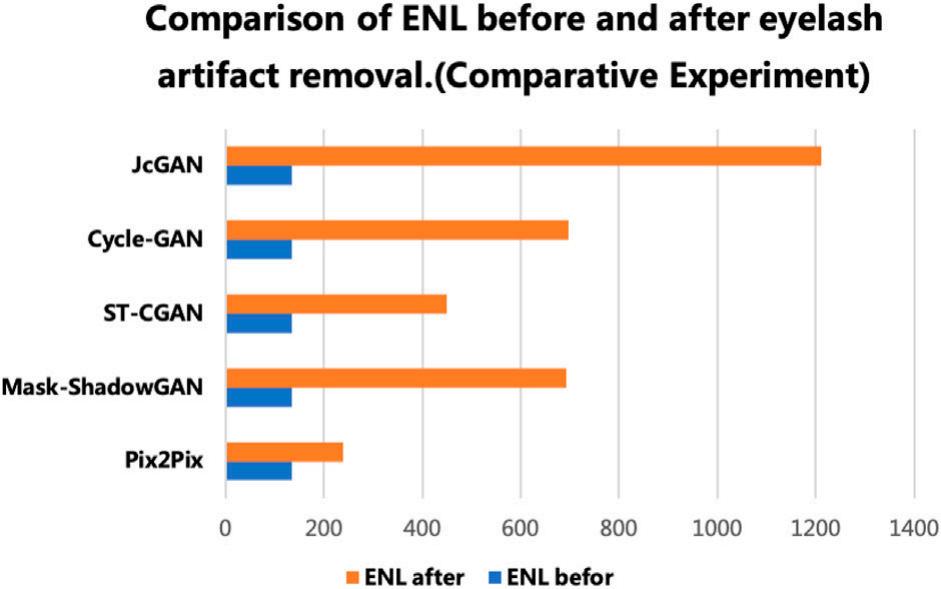
Comparison of ENL before and after the real eyelash artifact removal, where the blue bar represents the ENL value of the eyelash artifact area before eyelash artifact removal, and the orange bar represents the ENL value of the same area after eyelash artifact removal.

**FIGURE 9:**
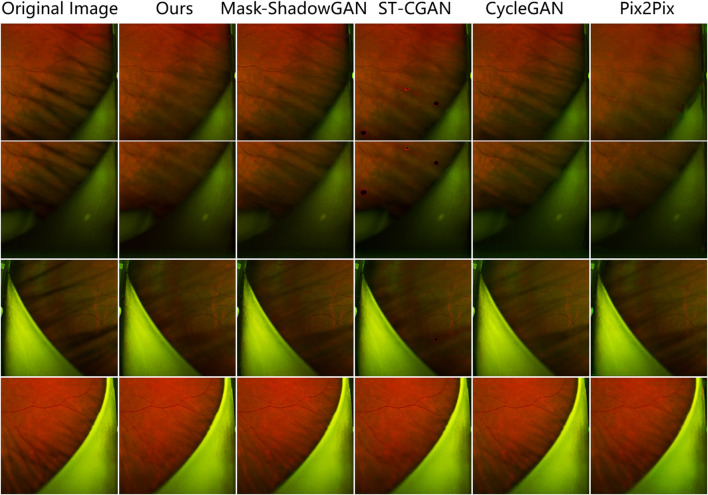
Comparison of the results of different methods on the uPRE Dataset. Our method removes the most eyelash artifacts and restores the most realistic background information.

The results show that our method achieves remarkable performance on the PSE Dataset. Our proposed JcGAN method achieves a high PSNR value of 43.6922 and a SSIM value of 0.9729, the highest among all methods. Compared with the best existing method, JcGAN improves PSNR and SSIM by 4.82% and 0.23%, respectively. At the same time, we compare the visual effects of the PSE Dataset test, and our method also achieves the best results, as shown in Figure 7. From Figure 7 we can see that our method achieves the best results in both eyelash artifact removal and background restoration. Compared with our method, none of the other methods completely remove eyelash artifacts. Among them, ST-CGAN has problems in the process of background recovery, which leads to information loss in the test image. Similarly, we perform the results validation of different methods on the uPRE dataset. We calculated the position ENL value of the local area of the eyelash artifact removal part for different methods. A comparison of ENL value results for different methods on the uPRE Dataset is shown in Figure 8. From Figure 8, we can see that our method JcGAN achieves the largest improvement in ENL value, which shows that our method restores the smooth background in the region removed for eyelash artifacts. Figure 9 shows a visual comparison of the results of different methods on the REL dataset.

### 6.2 Application to UWF image segmentation

**FIGURE 10:**
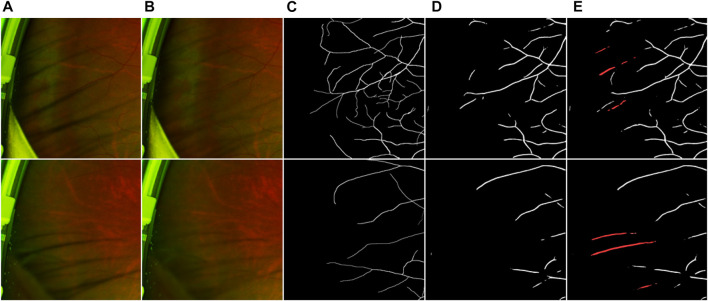
Vessel segmentation results. **(A)** Original image **(B)** Eyelash Removal image **(C)** Ground truth **(D)** The segmentation result of eyelash removal image **(E)** The segmentation result of original image. The red in **(E)** represents the wrong segmentation of eyelashes as blood vessels.

To verify that our proposed eyelash artifact removal algorithm can promote better processing and analysis of retinal vessels, a dedicated experiment for vessel segmentation in UWF images is performed. The corresponding segmentation results are shown in Figure 10, and sensitivity (SEN), Dice and area under curve (AUC) are shown in Table 5. ResU-Net Diakogiannis et al. (2020) is adopted as the segmentation network. 406 eyelash-free images are used to train ResU-Net. For the trained segmentation model, the original image with eyelashes and the image processed by JcGAN are used for testing respectively. Assessment via vessel segmentation illustrates that the SEN, Dice and AUC of ResU-Net have respectively increased by 3.64%, 1.54%, and 1.43% after eyelash artifact removal using JcGAN, as shown in Table 5. As shown in Figure 10; Table 5, the eyelash-removed images have better performance on the vessel segmentation task than the original images. The images processed by our JcGAN network successfully solved the problem that eyelashes were incorrectly segmented as blood vessels, and improved the overall segmentation performance.

**TABLE 5:** The results of the eyelashes removal image and the original image on the blood vessel segmentation index.

Methods	ResU-net		
	SEN	Dice	AUC
Original image	0.4663	0.5124	0.8783
Eyelash Removal image	0.4833	0.5203	0.8909

### 6.3 Summary

Artifacts caused by eyelash occlusions hinder high-quality inspection on retinopathy at wide range in UWF fundus images. In this work, we tackle the issue of eyelash artifacts existing in UWF fundus images with deep learning technique for the first time. We firstly collect UWF fundus images and construct two eyelash datasets called paired synthetic eyelashes (PSE) and unpaired real eyelashes (uPRE) respectively. Based on the two datasets, we have proposed a deep learning approach called Joint conditional Generative Adversarial Networks (JcGAN) to eliminate eyelash artifacts in UWF fundus images. The proposed JcGAN could jointly learn the mapping from images with real or synthetic eyelash artifacts to artifact-free ones via two generative adversarial networks with a shared generator. In addition, a background refinement module is trained with the generator in an end-to-end manner to further recover the detailed information of regions corrupted by eyelash artifacts. The experimental results on both PSE and uPRE dataset show that our eyelash artifact removal approach have achieved the best performance. Compared with other deep learning methods, our JcGAN can remove eyelash artifacts more effectively and achieve higher visual effect. Furthermore, JcGAN can significantly facilitate vessel segmentation in UWF fundus images due to the improved visibility of vessels obscured by eyelash artifacts. In the future, we will consider exploring a more appropriate method to construct paired synthetic eyelash samples and introducing prior knowledge of eyelash artifacts into the deep learning model. Furthermore, we will apply our approach to lesion segmentation tasks (e.g., identifying hemorrhages and exudates) as a preprocessing procedure to verify the effectiveness of eyelash artifact removal.

## Data Availability

The raw data supporting the conclusion of this article will be made available by the authors, without undue reservation.
